# Neuroprotective effects of paederoside against mitochondrial dysfunction in rotenone-induced cell models of Parkinson’s disease

**DOI:** 10.3389/fnins.2026.1853888

**Published:** 2026-06-18

**Authors:** Liping Sun, Juan Lang, Zhongkui Xiong

**Affiliations:** 1 Department of Pathology, Shaoxing People’s Hospital, Shaoxing, Zhejiang, China; 2 School of Medicine, Shaoxing University, Shaoxing, Zhejiang, China; 3 Department of Radiation Oncology, Shaoxing Second Hospital, Shaoxing, Zhejiang, China

**Keywords:** mitochondria, Neuro-2A cells, paederoside, Parkinson’s disease, rotenone

## Abstract

**Introduction:**

Parkinson’s disease (PD) is the second most prevalent neurodegenerative disorder and is one of the leading causes of neurological disability. Although there are currently no cures or treatments for this disease, many patients with PD may therapeutically benefit from preventing or mitigating mitochondrial dysfunction. This study aimed to investigate the protective effects of paederoside against rotenone-induced neuronal damage in Neuro-2A (N2A) cell models of PD.

**Methods:**

First, the cytotoxic effects of rotenone on N2A cells were assessed using the cell counting kit-8 assay to establish a cell model of PD. Second, the effects of paederoside on mitochondrial complex I activity, mitochondrial swelling, and caspase-3 activity in the rotenone-induced cellular model were investigated. Finally, the impact of paederoside on tyrosine hydroxylase-positive cells in a rotenone-induced co-culture of N2A cells and BV-2 cells (N2A/BV-2) was studied.

**Results:**

N2A cells were significantly damaged by rotenone in a time- and concentration-dependent manner; a 48 h rotenone treatment at 20 nM was used to create a cell model of PD. Pretreatment with 1 μM paederoside notably increased mitochondrial complex I activity in N2A cells with rotenone-induced impairment, leading to 42.85% increase. Additionally, pretreatment with 0.1, 1, and 10 μM paederoside considerably alleviated rotenone-induced mitochondrial swelling as indicated by the optical density (OD) (10 min)/OD(0 min) ratio, which increased by 1.21-, 1.33-, and 1.37-fold, respectively. Notably, pretreatment with 10 μM paederoside significantly inhibited caspase-3 activation triggered by rotenone in this cellular setting, resulting in a 39.93% decrease in enzyme activity. Moreover, pretreatment with 10 μM of paederoside markedly enhanced the number of TH-positive cells in a co-culture system consisting of N2A/BV-2 cells induced by rotenone, with increases of 82.79%.

**Conclusion:**

Pretreatment with paederoside may protect against mitochondrial dysfunction in cell models of PD induced by rotenone.

## 1 Introduction

Parkinson’s disease (PD) is the second most common neurodegenerative disorder and one of the main causes of neurological disability, affecting about 2% of people over 60 (Singh and Ganley, 2021; Tolosa et al., 2021). It is characterized by motor and non-motor symptoms due to a pathophysiologic loss or degeneration of dopaminergic neurons in the substantia nigra of the midbrain and the development of neuronal Lewy bodies (Beitz, 2014; Tysnes and Storstein, 2017). Mitochondrial dysfunction and oxidative stress are regarded as important factors in the dopaminergic neurodegeneration associated with PD (Jin et al., 2014). It is believed that dopaminergic neurons are particularly susceptible to mitochondrial dysfunction (Goiran et al., 2022). PD-like pathology may be initiated and propagated by damaged mitochondrial DNA (mtDNA) (Tresse et al., 2023). The mitochondrial abnormalities linked to the disease include an impaired electron transport chain, altered mitochondrial morphology and dynamics, mutations in mtDNA, and disrupted calcium homeostasis (Subramaniam and Chesselet, 2013). Mitochondrial dysfunction is a common characteristic of both sporadic and monogenic PD and may represent an early defect in disease pathogenesis (Subramaniam and Chesselet, 2013; Bose and Beal, 2016).

Currently, neither cures nor disease-modifying therapies are available (Gonzalez-Rodriguez et al., 2021). Developing novel treatment strategies may be guided by a better understanding of mitochondrial function in PD pathophysiology (Moradi Vastegani et al., 2023), and many patients with PD may benefit therapeutically from preventing or mitigating mitochondrial dysfunction (Ramsey and Giasson, 2007). Plant-derived iridoids, such as asperuloside, catalpol, harpagoside, and others, have been demonstrated to have marked neuroprotective effects and the capacity to slow neurodegeneration progression in PD (Dinda et al., 2019; Kushawaha et al., 2025). Asperuloside, a naturally occurring iridoid glycoside derived from medicinal plants, has demonstrated promise as a neuroprotective agent by modulating key signaling pathways, including restoring mitochondrial function and promoting neuronal survival (Kushawaha et al., 2025). Pretreatment with 0.5 mM catalpol, an iridoid, for 1 h before exposure to 1-methyl-4-phenyl-1,2,3,4-tetrahydropyridine (MPTP) successfully reduced mitochondrial dysfunction in mesencephalic neuron-astrocyte co-cultures (Bi et al., 2008). This protective effect was mediated by the restoration of mitochondrial complex I activity, stabilization of mitochondrial membrane potential (MMP), normalization of intracellular Ca^2+^ levels, reduction of reactive oxygen species (ROS) accumulation, and inhibition of mitochondrial permeability transition (MPT) pore opening (Bi et al., 2008). Another iridoid, harpagoside (≥0.1 μM), notably reduced rotenone-induced mitochondrial swelling, and 1 μM harpagoside successfully reversed the inhibitory effects of rotenone on complex I activity, indicating its protective effects against mitochondrial dysfunction (Lang and Xiong, 2025a).

The main components of two significant *Paederia* species (*Rubiaceae*), *Paederia foetida* and *Paederia scandens*, are iridoid glycosides, such as asperuloside, paederoside, and scanderoside (Ai et al., 2023; Dwivedi et al., 2025). *P. foetida* is used to treat inflammation, piles, and diarrhea in traditional medicine. On the contrary, *P. scandens* is used to alleviate aches, jaundice, dysentery, and dyspepsia (Wang et al., 2014; Li et al., 2019). Scopolamine-induced dementia that mimicked Alzheimer’s disease was considerably alleviated by pretreatment with 1,000 mg/kg *P. foetida* leaf extract (Pakaprot et al., 2024). Renal injury induced by uric acid nephropathy notably improved when treated with iridoid glycosides of *P. scandens*, and also inhibited the activity of nuclear factor-κB (NF-κB), p65, monocyte chemoattractant protein-1, and *α*-smooth muscle actin (Zhu et al., 2012). Paederosidic acid has been demonstrated to successfully alleviate oxidative stress and inflammation in lipopolysaccharide-treated rats. Paederosidic acid markedly upregulated the expression of superoxide dismutase 2 (SOD2) while concurrently downregulating the expression of tumor necrosis factor-*α* (Tao et al., 2024). *P. scandens (Lour.) Merr*. demonstrated hepatoprotective properties against acetaminophen-induced hepatic injury in rats by modifying inducible nitric oxide synthase (NOS) and NF-κB (Tang et al., 2022).

Rotenone, a classic mitochondrial complex I inhibitor, leads to the progressive death of dopaminergic neurons and *α*-synuclein inclusions in rats, in addition to destabilizing microtubules (Ibarra-Gutierrez et al., 2023). Our research group has identified that paederoside exerts protective effects against rotenone-induced neurotoxicity in Neuro-2A (N2A) cell models of PD, in part, by modulating the NF-κB/NOS/nitric oxide (NO)/*α*-synuclein nitration pathway (Lang and Xiong, 2025b). It has not yet been investigated whether paederoside exerts protective effects on cellular models of PD through mitochondrial regulation. This study examined whether paederoside exhibited mitochondrial protective effects in rotenone-induced N2A cell models.

## 2 Materials and methods

### 2.1 Drugs and reagents

**Figure 1:**
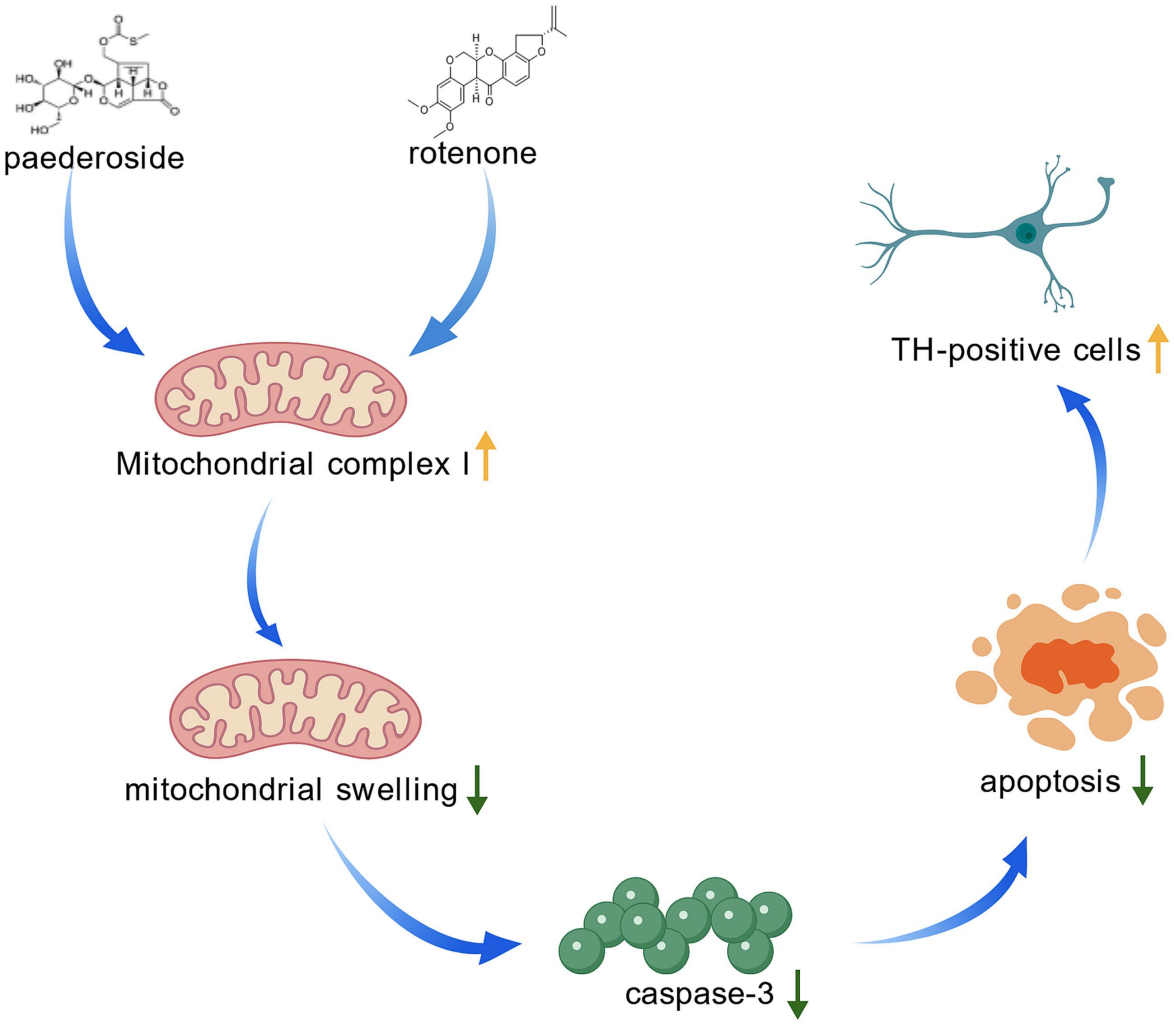
Schematic representation of the mechanism of action of the drug created with BioGDP.com. The results indicated that pretreatment with paederoside protects against mitochondrial dysfunction in rotenone-induced cell models of PD. We hypothesized that rotenone inhibits the activity of mitochondrial complex I, leading to mPTP opening, followed by mitochondrial swelling and caspase-3 activation. Lastly, this cascade induces apoptosis in TH-positive cells; however, this process is counteracted by paederoside.

Paederoside (Purity 98%) was acquired from Wuhu Delta Pharmaceutical Technology Co., Ltd., China (Figure 1). Rotenone and dibutyryl cyclic adenosine monophosphate (db-cAMP) were procured from Sigma-Aldrich. Dulbecco’s modified Eagle medium (DMEM), minimal essential medium (MEM), fetal bovine serum (FBS), and trypsin were acquired from Invitrogen. The mitochondria isolation kit and caspase-3 detection kit were procured from Beyotime Institute of Biotechnology. Nicotinamide adenine dinucleotide reduced (NADH), potassium ferricyanide, Triton X-100, and N-2-hydroxyethylpiperazine-N′-2-ethanesulfonicacid (HEPES) were acquired from Aladdin Industrial Corporation. The polyclonal antibody against tyrosine hydroxylase (TH) (Cat. No. AB152) was procured from Millipore. The secondary anti-rabbit/mouse antibodies (SA1020, Cat No. 08E25C), streptavidin–biotin complex (SABC), and 3,3′-diaminobenzidine (DAB) were acquired from Wuhan Boster BioEngineering Co., Ltd.

### 2.2 N2A cell differentiation

The N2A cell line was obtained from the Cell Bank of the Committee on Typical Culture Collection, Chinese Academy of Sciences. N2A cells were cultured in MEM supplemented with 10% FBS, 50 U/mL penicillin, and 50 μg/mL streptomycin, in a humidified atmosphere with 5% CO_2_ at 37 °C. N2A cells must be differentiated for use in PD research because they exhibit distinct oncogenic characteristics and lack certain properties unique to dopaminergic neurons. The concentration of FBS in the culture medium was reduced from 10 to 0.5% as reported in the literature (Tremblay et al., 2010), and 1 mM db-cAMP was added and retained for 3 d to promote differentiation. The differentiated N2A cells were used for subsequent experimental research.

### 2.3 BV-2 cell culture

The BV-2 cell line was acquired from Shanghai Fuxiang Biotechnology Co., Ltd. BV-2 cells were cultured in DMEM supplemented with 10% FBS, 50 U/mL penicillin, and 50 μg/mL streptomycin in a humidified environment with 5% CO_2_ at 37 °C.

### 2.4 N2A/BV-2 cell co-culture

Co-cultures of N2A and BV-2 cells (1,4) were cultured in a mixed solution of DMEM/MEM (1,1), 0.5% FBS, 1 mM db-cAMP, 50 U/mL penicillin, and 50 μg/mL streptomycin in a humidified environment with 5% CO_2_ at 37 °C.

### 2.5 Cell viability determined by cell counting kit-8 (CCK-8) assays

N2A cells were seeded at a density of 5 × 10^4^ cells/mL in 96-well plates (3.125 × 10^4^ cells/cm^2^, 200 μL per well) and allowed to adhere overnight. Then, rotenone was added. The final seven concentrations of rotenone were 0, 0.01, 0.1, 1, 10, 100, and 1,000 nM. Cell viability was evaluated at 0, 6, 24, 48, and 72 h after rotenone treatment. There were three replicate wells in each experimental group. Additionally, 10 μL of CCK-8 reagent was added to each well of the culture plate at a predetermined time point to assess cell viability. The cells were then incubated in a CO_2_ incubator for 2 h. Lastly, the samples were tested at an absorbance wavelength of 450 nm with a reference wavelength of 620 nm using a microplate reader. The experiment was performed three times, and the number of repeats served as the sample size for the study.

A cell model of PD was established for all experiments by treating cells with 20 nM rotenone, except for the assessment of mitochondrial complex I activity. The reasons were as follows. (i) Mitochondrial complex I activity assays were performed on isolated mitochondria instead of intact cells, whereas all other studies were conducted in intact cellular culture systems. (ii) Rotenone was administered for 10 min in the mitochondrial complex I activity assays, while its exposure was 9, 24, or 48 h in the other experiments. (iii) A concentration of 2.5 μM rotenone was used for mitochondrial complex I activity assays, selected based on the following rationale: 1 μM rotenone was administered to SH-SY5Y cells for assessing mitochondrial complex I activity (Li et al., 2022), while 2.5 μM rotenone was administered to N2A cells for assessing mitochondrial complex I activity (Lang and Xiong, 2025a).

### 2.6 Mitochondrial complex I activity assays

N2A cells were cultured for 48 h at a density of 5 × 10^4^ cells/mL in 25 cm^2^ cell culture flasks (5 mL/flask, 1 × 10^4^ cells/cm^2^). Then, 2 × 10^7^ cells were removed for mitochondrial extraction. The mitochondrial isolation process, as well as the specific methodologies and procedural steps for assessing mitochondrial complex I activity were detailed in reference (Lang and Xiong, 2025b). The experiment included four groups: a control group, a model group, two paederoside-treated groups (treatment with paederoside and rotenone). The final doses were 0.1 and 1 μM in the low- and high-dose groups, respectively. The mitochondrial complex I activity assay reaction system included 35 μL of detection buffer, 5 μL of mitochondrial lysate, 5 μL of coenzyme solution, and 5 μL of rotenone. The contents of the detection buffer were 0.17 mM NADH, 0.6 mM potassium ferricyanide, 1 mL/L Triton X-100, and phosphate buffer (pH 7.4). Mitochondria were added to initiate the reaction, and the absorbance of NADH was measured at 340 nm, 30 °C, and 10-min intervals using a spectrophotometer. Standard curves were prepared in parallel. Mitochondrial complex I activity was expressed as nmol of oxidized NADH per minute per mg of mitochondrial protein.

### 2.7 Mitochondrial swelling assays

The experimental groups included a control group, a model group, and four paederoside-treated groups (treatment with paederoside and rotenone). N2A cells were plated at a density of 1 × 10^6^ cells/mL (2.083 × 10^5^ cells/cm^2^) in 9.6 cm^2^ 6-well plates with a volume of 2 mL/well. Paederoside was added to final concentrations of 0.01, 0.1, 1, and 10 μM following a 12-h culture. Then, 20 nM rotenone was added 2 h after paederoside treatment, and the cells were cultured for an additional 9 h. Paederoside was maintained in the cell culture system throughout this period without any washout procedures, and the mitochondria were then isolated. The mitochondrial swelling assay was conducted according to the instructions (Lang and Xiong, 2025a; Huang et al., 2013). The first buffer, named A, consisted of 125 mM sucrose, 65 mM KCl, 10 mM HEPES/KOH (pH 7.4), and 5 mM potassium succinate, while the second buffer, named B, contained 10 mM CaCl_2_. The reaction mixture was composed of 50 μL of Buffer A, 10 μL of Buffer B, and 40 μL of the sample. Immediately after the addition of CaCl_2_, the absorbance was measured at 540 nm, with readings taken every 2 min for 10 min. Mitochondrial swelling was quantified based on the decrease in absorbance during this period.

### 2.8 Determination of caspase-3 activities

This experiment included four groups: a control group, a model group, and two paederoside groups (treatment with paederoside and rotenone). The final paederoside concentrations were 1 and 10 μM, for the low- and high-dose groups, respectively. N2A cells were seeded at a density of 2 × 10^5^ cells/mL (4.166 × 10^4^ cells/cm^2^) in 6-well plates, and paederoside was added after 12 h of cultivation. Additionally, 20 nM rotenone was added 2 h after paederose treatment, and the cells were further cultured for an additional 24 h. The cells were scraped with a cell scraper and centrifuged at 600 ×*g* for 5 min at 4 °C. The pellet was then resuspended in PBS, washed once, and centrifuged again under the same conditions to collect the cell precipitate. Then, the pellet was resuspended in 100 μL of cell lysis buffer and incubated on ice for 15 min to complete cell lysis. The lysate was spun at 16,000 ×*g* for 10 min at 4 °C, and the supernatant was collected for further caspase-3 activity assays. The caspase-3 activity assay was performed according to the manufacturer’s instructions. Enzyme activity was calculated as follows: one unit of caspase-3 activity was considered as the quantity of enzyme that catalyzed the conversion of 1 nmol of Ac-DEVD-pNA to pNA per hour at 37 °C.

### 2.9 Immunocytochemical staining

The groups established for the experiment included a control group, a model group, and paederoside intervention groups (with final concentrations of 0.01, 0.1, 1, and 10 μM). The co-culture of N2A/BV-2 cells was prepared using a volume of 200 μL/well and a total cell density of 5 × 10^4^ cells/mL (3.125 × 10^4^ cells/cm^2^). Paederoside was added after 12 h of cultivation. Additionally, 20 nM rotenone was added 2 h after paederoside exposure, and the cells were cultured for an additional 24 h. Cells were washed with PBS for 5 min, and the process was repeated three times. Endogenous peroxidase activity was terminated by incubating the cells with 3% H_2_O_2_ at room temperature for 20 min. After three additional PBS washes, they were treated with 0.3% Triton X-100 at room temperature for 30 min to increase membrane permeability, then washed three times more with PBS. Non-specific binding was removed by incubating in 5% BSA for 30 min at room temperature, followed by rinsing with PBS. The primary antibody (anti-TH, rabbit-derived, dilution 1:100) was added and incubated overnight at 4 °C, and then at 37 °C for 1 h. The primary antibody was replaced with 5% BSA as a negative control. The cells were rinsed three times with PBS for 5 min each. The secondary antibody (anti-rabbit/mouse IgG, 1:1) was added dropwise and incubated for an hour at room temperature, followed by three washes with PBS. SABC reagent was then added and incubated at room temperature for 30 min, followed by three washes with PBS. DAB chromogen and stop reaction were used to develop color after the desired staining intensity was achieved. Images/fields of view at 200 × magnification and 10 images per well were obtained for each group using a Nikon Eclipse TS100 inverted microscope. The cell number per field of view was manually counted. The cell number for each group is represented by the average number of cells counted.

### 2.10 Statistical analyses

All data are presented as mean ± standard error of the mean (SEM). Statistical analyses were performed using the Statistical Package for the Social Sciences software (version 18.0). One-way analysis of variance (ANOVA) was used for comparing multiple groups, followed by the Tukey *post hoc* test for pairwise comparisons when significant differences were detected. Student’s t-test was used to compare two groups. A *p*-value < 0.05 was considered statistically significant.

## 3 Results

### 3.1 Effects of rotenone on N2A cell viability

**Figure 2:**
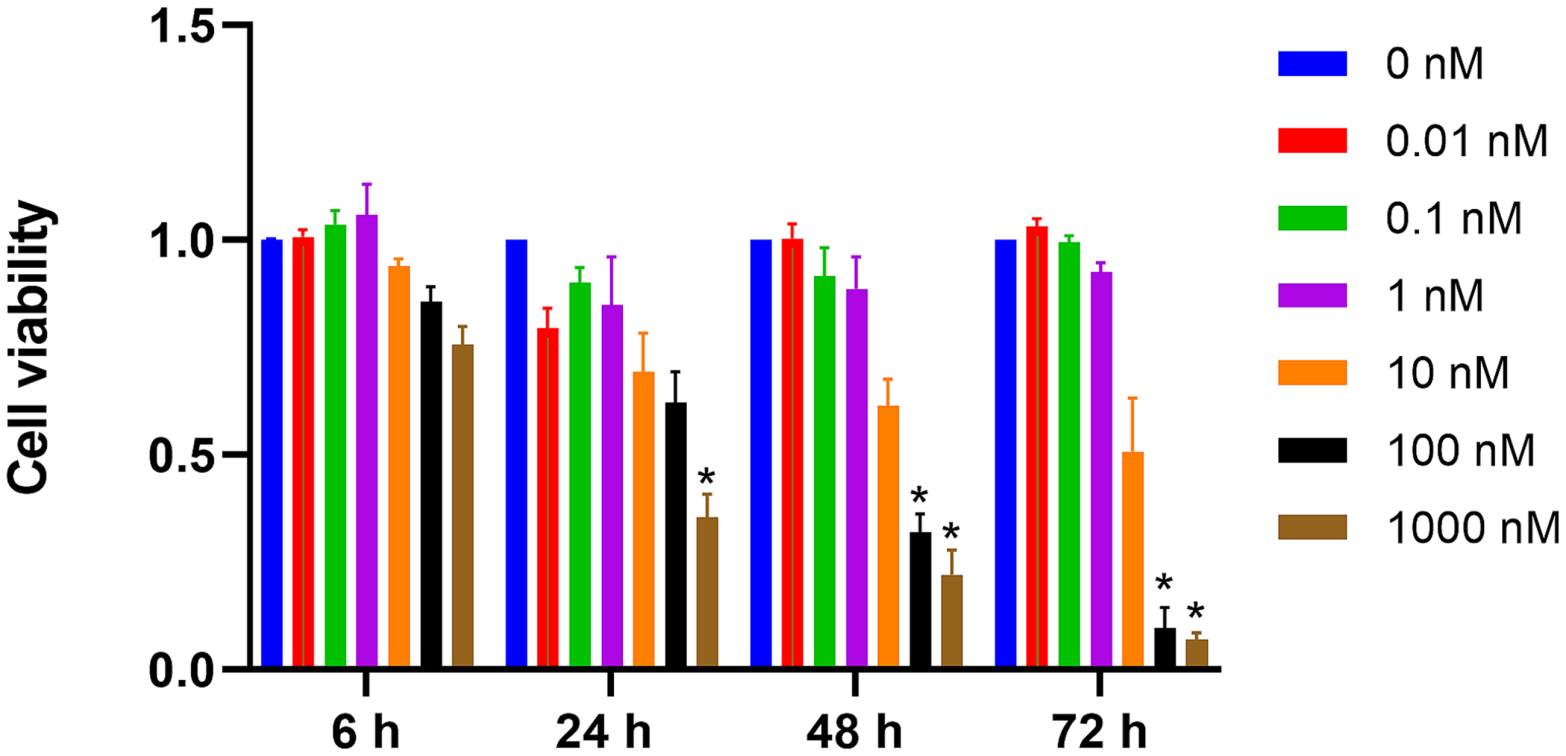
Effects of paederoside on cell viability. *p < 0.05 as compared with group treated with 0 nM rotenone for 6 h (*n* = 3).

Figure 2 presents that when the rotenone concentration was 100 nM, N2A cell proliferation was significantly inhibited after 48 h of exposure (*p* < 0.05, *p* < 0.05). A pronounced inhibitory effect was observed at rotenone concentration of 1,000 nM, with cell proliferation significantly suppressed as early as 24 h post-treatment (all *p* < 0.05). GraphPad Prism version 9.5.1 was employed to determine the 48-h half-maximal inhibitory concentration (IC_50_) of rotenone, which was calculated to be 13.2 nM. In conclusion, rotenone causes significant damage to N2A cells in a time- and concentration-dependent manner.

### 3.2 Effect of paederoside on rotenone-induced mitochondrial complex I (NADH dehydrogenase) activity in N2A cells

**Figure 3:**
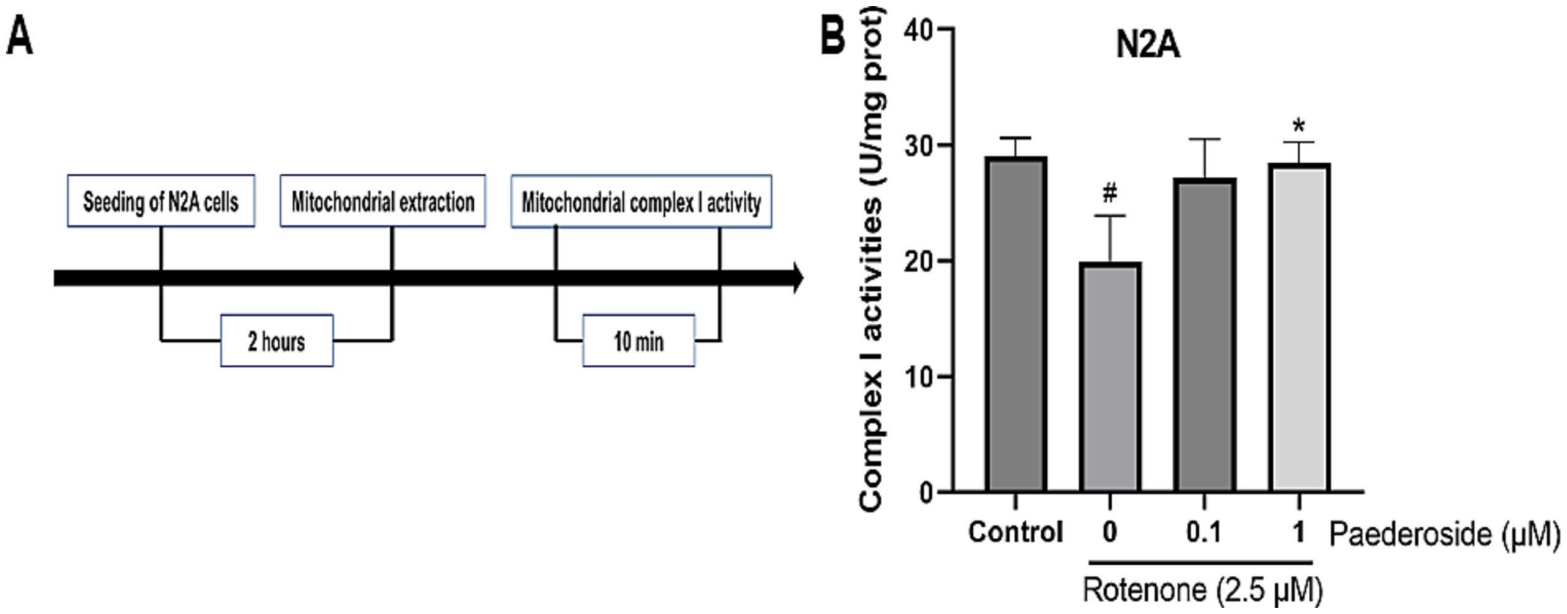
Mitochondrial complex I activity. **(A)** Protocol for treatments and examinations; **(B)** Effects of paederoside on mitochondrial complex I activities induced by rotenone. ^#^*p* < 0.05 as compared with control group, *p < 0.05 as compared with rotenone-treated group (*n* = 3).

Figure 3 demonstrates that exposure of 2.5 μM rotenone for 10 min significantly inhibited mitochondrial complex I enzyme activity in N2A cells. The enzyme activity decreased from 29.04 ± 0.92 U/mg (control group) to 19.93 ± 2.31 U/mg (model group), representing a 31.37% reduction (*p* < 0.05). The activity of complex I increased from 19.93 ± 2.31 U/mg to 28.47 ± 1.03 U/mg after pretreatment with 1 μM paederoside, representing increases of 42.85%, respectively (*p* < 0.05).

### 3.3 Effect of paederoside on rotenone-induced mitochondrial swelling in N2A cells

**Figure 4:**
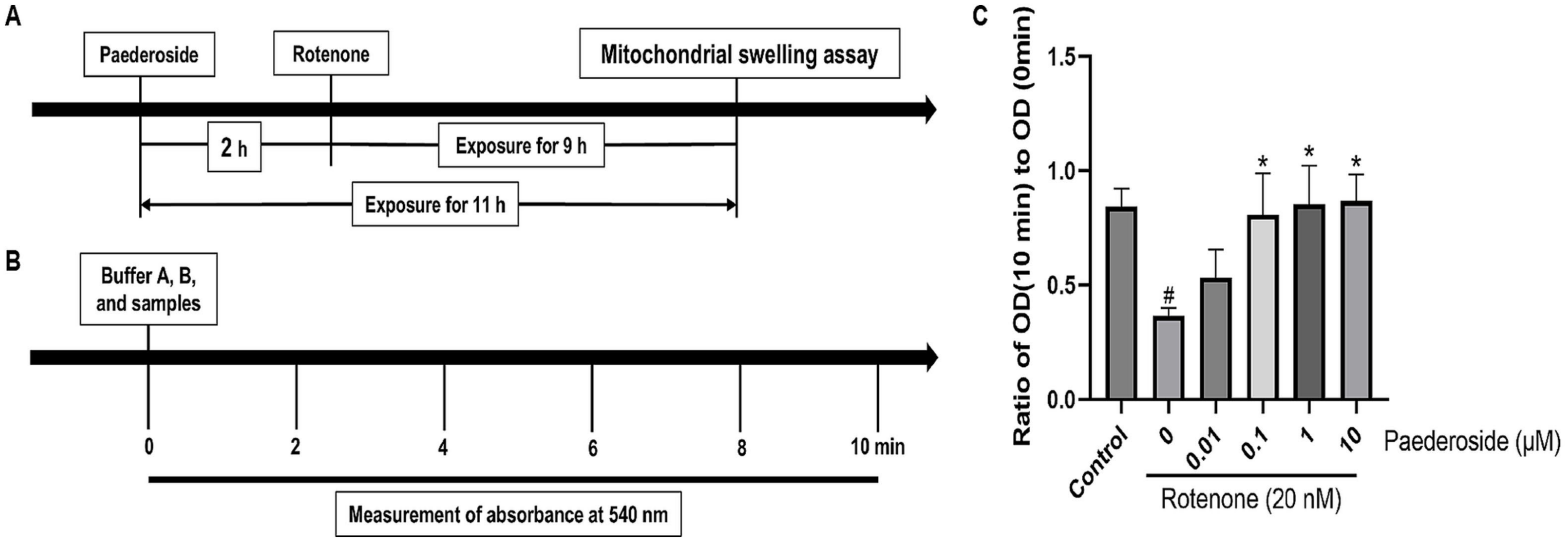
Mitochondrial swelling. **(A)** Protocol for treatments; **(B)** Protocol for examinations; **(C)** Effects of paederoside on mitochondrial swelling induced by rotenone. ^#^*p* < 0.05 as compared with control group, *p < 0.05 as compared with rotenone-treated group (*n* = 3).

CaCl_2_ solution was added to the mitochondrial detection system for the mitochondrial swelling assay (Figure 4). Absorbance at 540 nm decreased gradually as mitochondrial permeability increased, with a greater amplitude indicating greater swelling. The assay lasted 10 min, and the extent of mitochondrial swelling was quantified as the ratio of optical density (OD) at the end of the measurement period (10 min) to baseline (0 min). A lower ratio indicated greater mitochondrial swelling. The OD (10 min)/OD (0 min) ratio in the control group was 0.842 ± 0.046. This ratio significantly decreased to 0.366 ± 0.019 (*p* < 0.05). Pretreatment with 0.1, 1, and 10 μM paederoside for 2 h increased this ratio from 0.366 ± 0.019 to 0.808 ± 0.104, 0.852 ± 0.098, and 0.867 ± 0.067, which was 1.21-, 1.33-, and 1.37-fold increase, respectively (all *p* < 0.05).

### 3.4 Effect of paederoside on rotenone-induced caspase-3 activity in N2A cells

**Figure 5:**
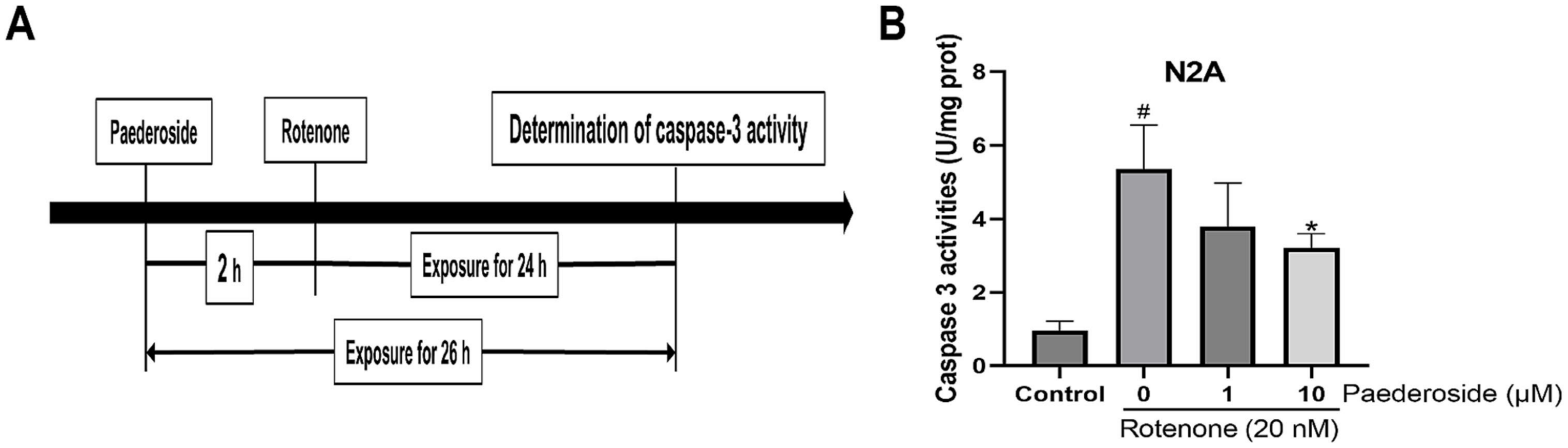
Caspase 3 activity. **(A)** Protocol for treatments and examinations; **(B)** Effects of paederoside on caspase 3 activities induced by rotenone. ^#^*p* < 0.05 as compared with control group, *p < 0.05 as compared with rotenone-treated group (*n* = 3).

Figure 5 indicates that the baseline caspase-3 activity in the control group was 0.95 ± 0.15 U/mg proteins. Caspase-3 activity increased markedly to 5.36 ± 0.69 U/mg protein (*p* < 0.05) after 24 h of rotenone treatment. Pretreatment with 10 μM paederoside for 2 h noticeably inhibited rotenone-induced caspase-3 activation, reducing its activity to 3.22 ± 0.22 U/mg protein, indicating a 39.93% reduction in enzyme activity (*p* < 0.05).

### 3.5 Effect of paederoside on the number of TH-positive cells in a rotenone-induced N2A/BV-2 cell co-culture system

**Figure 6:**
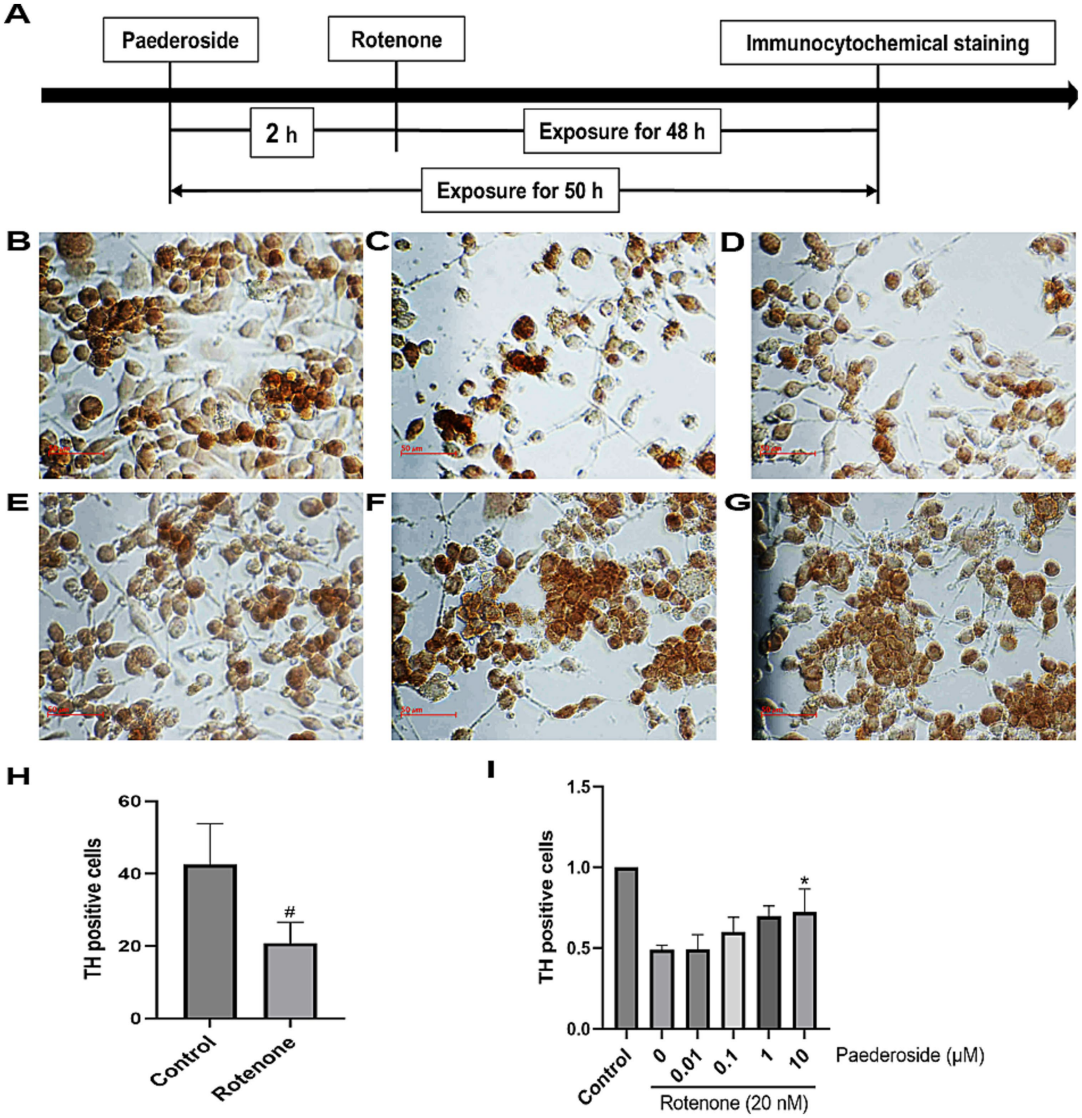
TH-positive cells identified using immunocytochemical staining. **(A)** Protocol for treatments and examinations; **(B)** Control group; **(C)** Model group (rotenone-treated group); **(D)** 20 nM rotenone + 0.01 μM paederoside; **(E)** 20 nM rotenone + 0.1 μM paederoside; **(F)** 20 nM rotenone + 1 μM paederoside; **(G)** 20 nM rotenone + 10 μM paederoside; **(H,I)** Effects of paederoside on TH-positive cells induced by rotenone. ^#^*p* < 0.05 as compared with control group, *p < 0.05 as compared with rotenone-treated group (*n* = 3).

TH staining of N2A/BV-2 cells was conducted using immunocytochemistry to examine dopamine neuron loss. Figure 6 illustrates that the number of TH-positive cells in the control group was 42.52 ± 13.81 cells/field of view. This number dropped to 21.28 ± 6.42 cells/field of view after rotenone induction (model group), indicating a substantial reduction of 51.2% (*p* < 0.05) (Figure 6H). The relative number of TH-positive cells in the model group was 0.488 ± 0.020 when compared to the control group, which was set at 1.000. Comparatively, the relative values obtained from the treatment group receiving 10 μM were 0.892 ± 0.067, indicating increases of 82.79% (*p* < 0.05) (Figure 6I).

## 4 Discussion

Paederoside is an iridoid glycoside, recognized as an active compound derived from *P. foetida* and *P. scandens*, which are two well-studied species within the *Paederia* genus (*Rubiaceae*) (Wang et al., 2014). An extract of *P. scandens (LOUR.) MERRILL (Rubiaceae)* demonstrates anti-inflammatory properties by regulating the production of pro-inflammatory mediators in synovial tissue and inhibiting the NF-κB signaling pathway (Ma et al., 2009). Iridoid glycosides from *P. scandens* represent important active components isolated from the traditional Chinese herb *P. scandens (LOUR) MERRILL*, belonging to the *Rubiaceae* family (Liu et al., 2012). Rats treated with total iridoid glycosides from *P. scandens (Lour.) Merr.* var. *tomentosa (Rubiaceae)* demonstrated a significant increase in glutathione, catalase, and SOD activity when compared to the control group, whereas malondialdehyde levels were reduced, and liver injury caused by 10% carbon tetrachloride was also inhibited (Peng et al., 2015). Pretreatment with 1,000 mg/kg *P. foetida* leaf extract notably mitigated the neurotoxic effects of scopolamine on rats (Pakaprot et al., 2024). The antioxidant activity of the extracts from *P. foetida Linn.* leafs was demonstrated by their ability to inhibit lipid peroxidation and enhance SOD, catalase, and glutathione peroxidase (Kumar et al., 2014). An essential oil of *P. scandens* markedly decreased hepatic ROS and malondialdehyde levels in a chicken non-alcoholic fatty liver disease model (Wu et al., 2019). Paederosidic acid has been demonstrated to increase *γ*-aminobutyric acid levels in the brain while decreasing glutamic acid concentrations (Yang et al., 2013).

Mitochondria are essential for the production of cellular energy, the regulation of calcium homeostasis, and the control of programmed cell death (Rani and Mondal, 2020). The activities of individual mitochondria within mitochondrial populations are expected to exhibit variability across different biological tissues (Khmelinskii and Makarov, 2021a). Mitochondrial impairment and oxidative stress are identified as key neuropathogenic mechanisms, which are supported by the identification of PD-associated genes involved in mitochondrial function (Imbriani et al., 2022). Mitochondrial dysfunction, a critical pathophysiological feature in the onset and progression of PD, leads to the accumulation and oligomerization of *α*-synuclein, whereas increased α-synuclein levels contribute to mitochondrial impairment (Prasuhn and Bruggemann, 2021; Di Maio et al., 2016). PD pathophysiology has long been linked to mitochondrial dysregulation, which includes deficiencies in mitochondrial complex I, increased oxidative stress, disruptions in mitochondrial dynamics, and reduced mitophagy (Choong and Mochizuki, 2023). Mitochondrial dysfunction in neurons is a primary factor contributing to the pathogenesis of incurable PD (Zheng et al., 2023). Addressing mitochondrial dysfunction is important for improving treatment approaches (Zheng et al., 2023). This study revealed that paederoside exhibited neuroprotective effects on rotenone-induced N2A cell models linked to PD by modulating mitochondrial function. This study revealed that lowering the FBS concentration in the culture medium from 10 to 0.5% and adding 1 mM db-cAMP enhanced the differentiation of N2A cells, which facilitated their application in PD research. Serum deprivation in combination with db-cAMP significantly enhances the differentiation of dopaminergic neurons from N2A (Tremblay et al., 2010). In the presence of db-cAMP, Western blotting and immunocytochemistry revealed a significant increase in TH expression, accompanied by elevated dopamine levels as quantified by high-performance liquid chromatography (Tremblay et al., 2010). Immunocytochemical staining indicated that the control group had 42.52 ± 13.81 TH-positive cells per field of view, suggesting that differentiated N2A cells express TH and exhibit traits typical of dopaminergic neurons. In this study, rotenone was used to develop N2A cell models that accurately mimic PD. N2A cell proliferation was considerably reduced after 24 h of exposure when the rotenone concentration exceeded 10 nM. Additionally, according to immunocytochemical staining results, the number of TH-positive cells significantly decreased after rotenone exposure. The results indicated that pretreatment with 1 and 10 μM of paederoside markedly increased the number of TH-positive cells in a co-culture system of N2A/BV-2 cells exposed to rotenone. TH is a rate-limiting enzyme expressed in various regions of the brain, but is particularly prominent in the midbrain. It plays a pivotal role in the biosynthesis of catecholamines including dopamine in dopaminergic neurons (Bhaskar et al., 2025). TH-immunoreactive neurons, notably located in the substantia nigra and ventral tegmental area, predominantly comprise dopamine neurons (Chu et al., 2025). Subacute rotenone treatment has been revealed to induce the loss of TH-positive neurons in the substantia nigra of rat models (Xiong et al., 2015).

Dichlorodihydrofluorescein fluorescence indicated a 58% increase in ROS production in rat pheochromocytoma PC-12 cells treated with 2 μM rotenone for 5 h. PC-12 cells treated with 2 μM rotenone for 24 h indicated a marked loss of MMP (60.76 ± 8.17%). Additionally, the caspase-3/7 assay revealed that PC-12 cells treated with 2 μM rotenone for 48 h displayed caspase-3/7 activity increase exceeding 500% (Li et al., 2012). Increased mitochondrial production of ROS promotes intracellular localization of the pro-apoptotic protein Bax to the outer membrane of mitochondria (Fiskum et al., 2003). In a previous study, we observed that rotenone induces the production of reactive nitrogen species (Lang and Xiong, 2025b). Moreover, 20 nM rotenone was used to establish PD cell models in our study, except for the mitochondrial complex I activity assay, which used 2.5 μM rotenone.

Mitochondrial complex I deficiency is observed in the substantia nigra of individuals diagnosed with PD (Flones et al., 2018) and is a defining characteristic of the disease (Gonzalez-Rodriguez et al., 2021). Idiopathic PD exhibits heterogeneous pathophysiology and can be categorized based on the severity of neuronal respiratory complex I deficiency, leading to two distinct subtypes with unique molecular and clinical characteristics (Flones et al., 2024). The mitochondrial complex I-deficient subtype accounts for about one-fourth of cases and is characterized by widespread neuronal complex I deficiency, a specific gene expression profile across different cell types, and an increased burden of neuronal mtDNA deletions (Flones et al., 2024). Mitochondrial complex I deficiency is actually enough to induce parkinsonism (Vos, 2022). Rotenone caused the redistribution of dopamine from vesicles to the cytosol in human dopaminergic SH-SY5Y cells (Watabe and Nakaki, 2007). This phenomenon may contribute to apoptosis of dopaminergic cells triggered by rotenone (Watabe and Nakaki, 2007). The mechanisms underlying neuronal cell death in PD are particularly characterized by a decrease in complex I activity (Kilbride et al., 2021). Mitochondrial complex I is an essential cellular energy transducer located within the inner mitochondrial membrane. The inhibition of complex I by rotenone depends on the flexibility of its ligand (Pereira et al., 2023). Rotenone-treated rats exhibited mitochondrial complex I syndrome, which was characterized by a 43 to 57% decrease in striatal complex I activity. This condition was linked to cellular oxidative stress observed in the dopaminergic regions of the striatum and frontal cortex (Valdez et al., 2019). The results of this study revealed that pretreatment with 1 μM paederoside significantly reduced the rotenone-induced impairment of mitochondrial complex I activity in N2A cells. Reversible swelling is associated with reversible inner mitochondrial membrane deformations, whereas irreversible swelling corresponds to irreversible deformations that may ultimately lead to membrane disruption (Khmelinskii and Makarov, 2021b). Mitochondrial swelling may be a contributing factor to the impaired transport of organelles in neuronal processes (Kaasik et al., 2007). The results demonstrated that pretreatment with 0.1, 1, and 10 μM paederoside markedly reduced rotenone-induced mitochondrial swelling in N2A cells. Mitochondrial swelling is a characteristic feature of mitochondrial dysfunction and serves as an indicator of the opening of the MPT pore (mPTP) (Gerencser et al., 2008). The initiation of mPTP results in the production of ROS at mitochondrial complex I (Batandier et al., 2004). The permeability transition pore is a channel in the inner mitochondrial membrane that is sensitive to Ca^2+^ levels (Chauvin et al., 2001). MMP assessment in peripheral blood mononuclear cells may serve as an early indicator of apoptosis in PD (Qadri et al., 2018). Mitochondrial membrane permeability transition may be involved in apoptosis induction by rotenone in liver cells (Isenberg and Klaunig, 2000). Dopamine oxidation alters mitochondrial respiration and triggers a permeability transition in brain mitochondria, with important consequences for PD (Berman and Hastings, 1999). The permeability transition pore in PD opens when *α*-synuclein oligomers interact with adenosine triphosphate synthase (Ludtmann et al., 2018). The apoptotic cell death of cerebellar granule neurons occurs through the mPTP opening, which is followed by swelling-induced rupture of the outer membrane of the mitochondria (Wigdal et al., 2002).

It has been reported that caspase-3 is activated by decreased NAD^+^ levels and a lower NAD^+^/NADH ratio, both of which are caused by a drop in mitochondrial complex I activity. This activation subsequently initiates the apoptotic process in granulosa cells (Wang et al., 2025). Caspase-3 activation is one of the essential features of PD, which causes neuron death through apoptosis and microglia activation through inflammation (Liu et al., 2013). The findings of this study revealed that pretreatment with 10 μM paederoside significantly inhibited caspase-3 activation induced by rotenone in N2A cells. The activation of caspase-3-mediated apoptosis in dopaminergic neurons may greatly influence the early pathogenesis of PD (Yamada et al., 2010). It causes apoptosis when highly activated, but at lower activation levels, it affects physiological processes, including corticostriatal long-term depression (Imbriani et al., 2019). It has been verified that rotenone-induced apoptosis is mediated by caspase 3 activity by increasing mitochondrial ROS production (Li et al., 2003). Caspase-3 regulates synaptic plasticity at corticostriatal synapses in the phosphatase and tensin homolog (PTEN) induced kinase 1 (PINK1) mouse model of PD (Imbriani et al., 2019). Rotenone inhibited the activity of vesicular monoamine transporter 2 (VMAT2), which is in charge of the uptake of dopamine into vesicles. The nitration of tyrosine residues in VMAT2 induced by rotenone causes both functional inhibition and the formation of aggregate-like structures of VMAT2 (Watabe and Nakaki, 2008). As a result, this leads to the redistribution of dopamine into the cytosol and triggers apoptosis in dopaminergic SH-SY5Y cells (Watabe and Nakaki, 2008). These findings revealed that rotenone-induced reduction of mitochondrial complex I activity was ameliorated by pretreatment with 1 μM paederoside, whereas increasing the dose of paederoside to 10 μM alleviated the rotenone-induced reduction of caspase 3 activity, indicating that paederoside has a lower threshold for mitochondrial complex I activity than for caspase 3 activity.

In this study, the N2A/BV-2 co-culture system was used to examine the protective effect of paederoside on TH-positive cells in a rotenone-induced cellular model for multiple reasons. It was reported that only 19% of all neurons are located in the cerebral cortex, despite its larger size, which accounts for 82% of the total human brain mass (Azevedo et al., 2009). The neuron-to-glia ratio in the human brain was modeled in this study using a mixed culture system with a N2A: BV-2 ratio of 1: 4. In Meda et al. (1995) used co-cultures of primary rat neuronal and N9 microglial cells to study Alzheimer’s disease. Another study used 2.5 × 10^5^ neurons per well and 2.5 × 10^5^ microglial cells per well to establish neuronal/microglial cell co-cultures (Ciesielski-Treska et al., 1998). Rotenone-induced mitochondrial dysfunction is associated with increased production of free radicals. Microglia-derived free radicals play a crucial role in PD pathogenesis, as demonstrated in disease models. BV-2 cells serve as microglial cells in the N2A/BV-2 co-culture system, whereas N2A cells represent TH-positive neurons. The decline in TH-positive neurons reflects the neurotoxic effects of rotenone-induced mitochondrial impairment and recapitulates important pathological features of PD in this cellular model. Pretreatment with paederoside reduced the rotenone-induced loss of TH-positive cells, suggesting its neuroprotective potential in the PD cell model. Pretreatment with 1 or 10 μM paederoside markedly attenuated the production of nitrated *α*-synuclein in response to rotenone exposure (Lang and Xiong, 2025b). Additionally, pretreatment with 10 μM paederoside significantly increased cell viability in rotenone-treated N2A cells (Lang and Xiong, 2025b).

According to our earlier research, paederoside was demonstrated to exert neuroprotective effects by modulating the NF-κB/NOS/NO/nitrated *α*-synuclein signaling pathway (Lang and Xiong, 2025b). Nitrative insult may initiate pathogenesis of *α*-synuclein aggregates (Paxinou et al., 2001). Under pathological conditions, mitochondrial accumulation of aggregated *α*-synuclein not only impairs Complex I activity, thereby increasing ROS production and triggering mitophagy but also disrupts numerous essential mitochondrial functions (Risiglione et al., 2021). Disruption of mitochondrial function and the consequent bioenergetic deficit represents an early, proximal event in the pathological cascade triggered by α-synuclein pathology in dopaminergic and cholinergic neurons at-risk in PD (Geibl et al., 2024). NF-κB essential modulator (NEMO), an NF-κB effector molecule, is recruited to damaged mitochondria in a Parkin-dependent manner (Harding et al., 2023). Mitochondrial dysfunction contributes to the accumulation and oligomerization of α-synuclein, which are key pathological features in PD (Di Maio et al., 2016). Accumulation of α-synuclein and mitochondrial dysfunction are both well-established pathological hallmarks of PD, and accumulating evidence supports a bidirectional pathogenic relationship between them (Di Maio et al., 2016). Rotenone-induced complex I deficiency triggers apoptosis by a mechanism that involves early nuclear translocation of NF-κB and activation of caspase-3 and MMP dissipation in SH-SY5Y cells (Wang et al., 2002; Condello et al., 2011). Mitochondria, mitogen-activated protein kinases, and NF-κB interact with each other in cellular models of PD, in which acute inhibition of complex I was induced by 1-methyl-4-phenylpyridinium ion in SH-SY5Y neuroblastoma cells (Cassarino et al., 2000). The mitochondrial protective effect of paederoside in the rotenone-induced cellular model may be attributed to its modulation of NF-κB signaling; however, further experimental validation is needed in subsequent investigations.

In conclusion, this study indicated that pretreatment with paederoside may protect against mitochondrial dysfunction in rotenone-induced cell models of PD. It was hypothesized that rotenone inhibits mitochondrial complex I activity, thereby probably triggering a cascade of downstream events, including mPTP opening, subsequent mitochondrial swelling, and activation of caspase-3. Ultimately, this cascade may induce apoptosis in TH-positive cells; however, this process could be attenuated or reversed by paederoside (Figure 1). This study undoubtedly has several limitations. (i) The current investigation was only conducted in an *in vitro* cellular model, without validation in *in vivo* animal experiments. (ii) A concentration of 2.5 μM rotenone significantly inhibited Complex I activity in isolated mitochondria; however, the effect of 20 nM rotenone on Complex I activity in intact cells remains undetermined. (iii) Despite the plausibility of the proposed mechanism (complex I inhibition → mPTP opening → caspase-3 activation → apoptosis), there is still insufficient evidence to support it. This is because important mechanistic markers, including coenzyme Q10 and cytochrome C release, MMP changes, and direct assessments of mPTP opening, have not been examined. Consequently, more experimental studies designed to address the aforementioned research questions are required to validate this hypothesis. Subsequently, we will corroborate our findings through animal studies and further detailed investigation into the mechanistic aspects.

## Data Availability

The raw data supporting the conclusions of this article will be made available by the authors, without undue reservation.
